# The causal relationship between human blood metabolites and risk of peripheral artery disease: a Mendelian randomization study

**DOI:** 10.3389/fcvm.2024.1435106

**Published:** 2024-09-10

**Authors:** Zhiyong Dong, Qingyun Wang

**Affiliations:** Department of Cardiothoracic Surgery, Beijing Shunyi Hospital, Beijing, China

**Keywords:** peripheral artery disease, blood metabolites, Mendelian randomization, biomarkers, GWAS

## Abstract

**Background:**

Peripheral Artery Disease (PAD) is a common vascular disorder typically caused by atherosclerosis, leading to impaired blood supply to the lower extremities, resulting in pain, necrosis, and even amputation. Despite extensive research into the pathogenesis of PAD, many mysteries remain, particularly regarding its association with human blood metabolites.

**Methods:**

To explore the causal relationship between 1,400 serum metabolites and PAD, a two-sample Mendelian randomization (MR) analysis was conducted. The Inverse Variance-Weighted (IVW) method was the primary technique used to estimate the causal impact of the metabolites on PAD. To enhance the analysis, several additional methods were employed: MR-Egger regression, weighted median, simple mode, and weighted mode. These methods provided a comprehensive evaluation beyond the primary IVW estimation. To ensure the validity of the MR findings, sensitivity analysis was performed. Furthermore, a bidirectional MR approach was applied to explore the possibility of a reverse causal effect between PAD and potential candidate metabolites.

**Results:**

After rigorous selection, significant associations were found between 1-(1-enyl-stearoyl)-2-arachidonoyl-GPE (p-18:0/20:4) and X-17653 levels with PAD. 1-(1-enyl-stearoyl)-2-arachidonoyl-GPE (p-18:0/20:4) was positively associated with increased PAD risk (IVW OR = 1.13, 95% CI, 1.06–1.21; *P* < 0.001). X-17653 levels were associated with decreased PAD risk (IVW OR = 0.88, 95% CI, 0.83–0.94; *P* < 0.001). In the reverse direction, PAD was positively associated with increased 1-(1-enyl-stearoyl)-2-arachidonoyl-GPE (p-18:0/20:4) levels (IVW OR = 1.16, 95% CI, 1.01–1.34; *P* = 0.036). PAD was not associated with X-17653.

**Conclusion:**

Among 1,400 blood metabolites, 1-(1-enyl-stearoyl)-2-arachidonoyl-GPE (p-18:0/20:4) and X-17653 are signiﬁcantly associated with PAD risk. Importantly, in the reverse direction, PAD was found to be positively associated with increased levels of 1-(1-enyl-stearoyl)-2-arachidonoyl-GPE (p-18:0/20:4). This highlights the bidirectional nature of the association and suggests a potential feedback mechanism between PAD and this specific lipid species. 1-(1-enyl-stearoyl)-2-arachidonoyl-GPE (p-18:0/20:4) may serve as potential biomarkers for PAD, aiding early diagnosis and providing novel avenues for personalized treatment and management. However, further validation and research are warranted despite the promising results.

## 1 Introduction

Peripheral artery disease (PAD) represents a prevalent vascular ailment primarily instigated by atherosclerosis, precipitating compromised blood perfusion to the lower extremities (1), consequentially yielding symptoms of ischemic pain, necrosis, and, in severe cases, necessitating limb amputation. Beyond encumbering patients’ daily routines, PAD escalates susceptibility to cardiovascular adversities such as myocardial infarction and stroke (2), profoundly impinging upon their overall well-being. Despite exhaustive inquiry into the pathophysiology of PAD, numerous enigmas persist, particularly concerning its nexus with human metabolic entities.

Human metabolites assume pivotal roles in sustaining vital activities and modulating physiological processes. In recent years, mounting empirical substantiation (3, 4) has underscored the intimate correlation between specific blood-borne metabolites and the etiology and progression of cardiovascular disorders. Some investigations have posited that metabolites influence the inception and advancement of diseases and emerge as plausible targets for therapeutic modalities (5, 6). Nevertheless, comprehension of the causative interplay between blood metabolites and PAD remains circumscribed.

To elucidate the intricate interplay between blood metabolites and PAD, this study employs the Mendelian randomization (MR) technique (7), a robust framework leveraging genetic variants to infer causality (8). Through amalgamating extensive genetic repositories with clinical datasets, our endeavor is geared towards unveiling latent causal connections between distinct blood metabolites and PAD, thereby furnishing novel theoretical underpinnings and clinical directives for the amelioration and management of PAD.

This exposition delineates the intricacies of our research blueprint, methodological approaches, and salient discoveries while underscoring the import of these revelations in deciphering the pathogenic mechanisms underpinning PAD and charting trajectories for clinical interventions.

## 2 Materials and methods

### 2.1 Research design

**Figure 1:**
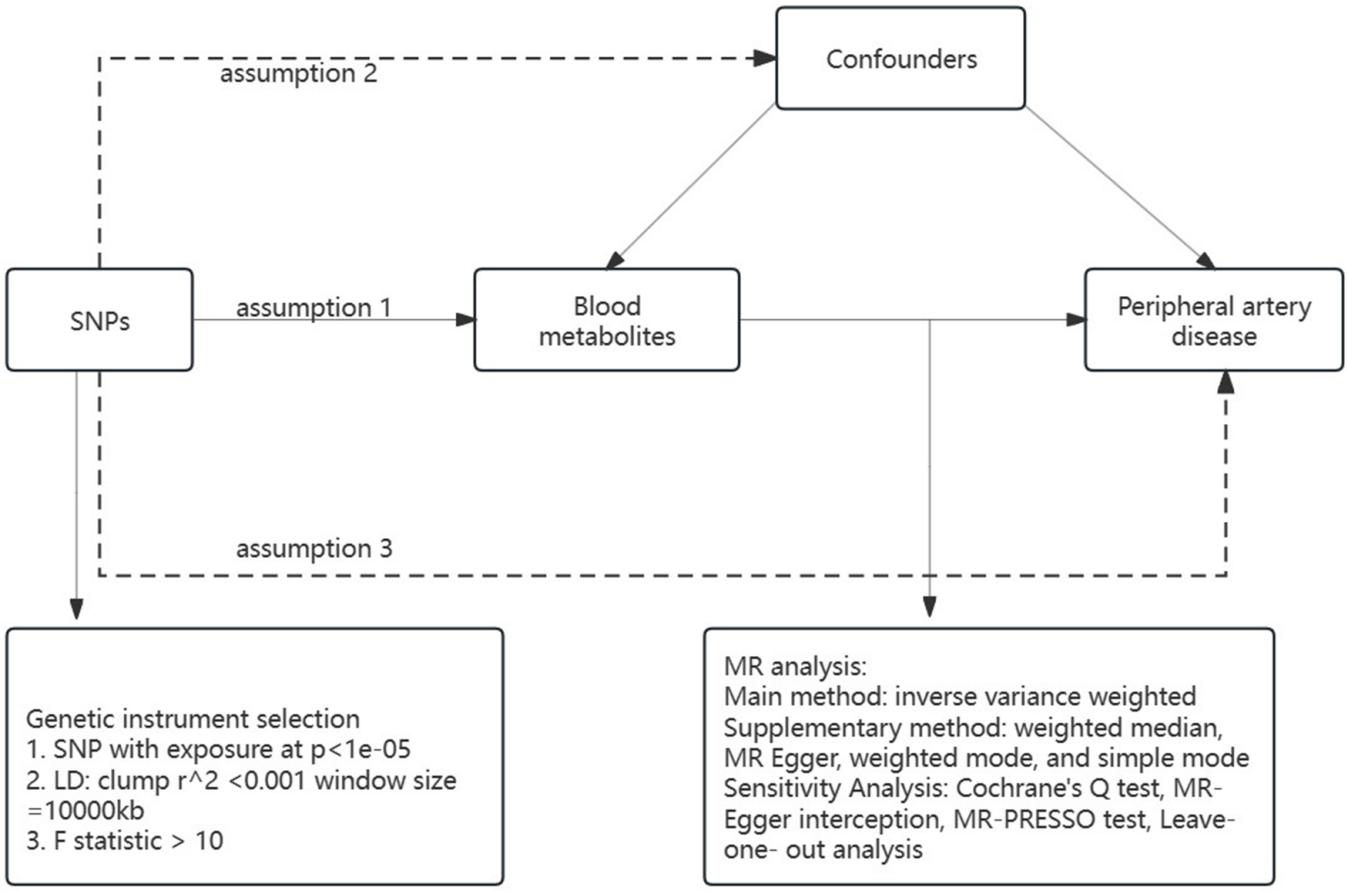
A summary of MR analysis and its three main hypotheses.

The research utilized a two-sample Mendelian randomization (MR) framework to methodically assess the causal link between 1,400 blood metabolites and the risk of PAD. Single nucleotide polymorphisms (SNPs) served as instrumental variables to mitigate the influence of confounders. The MR approach was grounded in three core principles: (1) relevance assumption, there must be a significant correlation between the SNPs and the exposure variable; (2) independence assumption: the SNPs should not be associated with any confounding variables; (3) exclusion restriction assumption, the SNPs are presumed to influence the outcome solely through the exposure variable. The study's adherence to these assumptions ensures the integrity of the causal inferences drawn from the MR analysis (Figure 1).

We employed a bidirectional Mendelian randomization (MR) approach, assessing both the effect of metabolites on PAD and the effect of PAD on potential candidate metabolites.

### 2.2 Data source

Previously, Shin and colleagues delved into the genetic determinants of human metabolism through a genome-wide association study (GWAS) focusing on untargeted metabolomics. The study encompassed 7,824 individuals from two distinct European cohorts. This extensive research led to the identification of 486 metabolites that exhibit genetic associations with human serum metabolite levels. Post rigorous quality control protocols, these 486 metabolites were further categorized into 309 known and 177 unknown entities (9). More recently (5), a comprehensive GWAS scrutinized 1,091 blood metabolites alongside the ratios pertaining to the aforementioned 309 metabolites. For those interested in exploring this data, it is readily available in the GWAS Catalog database. The entries for the extensive list of 1,400 blood metabolites are cataloged under the numbers GCST90199621 to GCS90201020.

The information on PAD was derived from a genome-wide association study (GWAS) spearheaded by Sakaue and colleagues in 2021. This extensive analysis included a cohort of 7,114 individuals diagnosed with PAD alongside 475,964 control subjects. The study meticulously analyzed over 24.18 million Single Nucleotide Polymorphisms (SNPs). A key ethical aspect of the original research was obtaining informed consent from every participant involved (10). The dataset is accessible online at the GWAS database (GWAS ID: ebi-a-GCST90018890).

### 2.3 Instrumental variable selection

We used standardized selection criteria to examine genetic variations among 1,400 metabolites. Understanding the possible constraints due to the limited number of SNPs achieving full genome-wide significance for metabolites, we adjusted the threshold by establishing a *p*-value of less than 1 × 10^−5^ (11). Upon identifying significant SNPs corresponding to each metabolite, we conducted a linkage disequilibrium analysis, considering LD to be present if the LD parameter *r*^2^ was <0.001 and the SNP distance was within 10,000 kb (12). Furthermore, to mitigate bias stemming from weak instrumental variables, we calculated the *F*-value for each SNP, designating SNPs with an *F*-value <10 as weak instruments (13).

### 2.4 MR analysis

In this study, the Inverse Variance-Weighted (IVW) method (14) served as the primary approach for estimating the causal relationship between metabolites and PAD (with a significance threshold of *p* < 0.05). The IVW method computes weighted estimates by leveraging the inverse of variances, assuming that all instrumental variables are valid. Specifically, it combines the Wald ratio associated with each Single Nucleotide Polymorphism (SNP) to derive a summary estimate (15). Despite its prominence in Mendelian randomization (MR) studies, it's essential to acknowledge that unknown confounding factors may still introduce genetic pleiotropy and bias when estimating effect sizes.

We employed several additional MR analysis methods, including weighted median (16), MR Egger (17), weighted mode (18), and simple mode (19), to complement our primary analysis. Heterogeneity was assessed using the Cochran *Q*-test (20), where a Cochran-Q derived *p*-value < 0.05 and *I*² > 25% indicated the presence of heterogeneity. To ascertain the stability of our findings, we conducted an analysis to see if the removal of any SNP would alter the results, thereby indicating the influence of a specific SNP (21, 22). This step is crucial to ensure that no individual SNP disproportionately affects the outcome. Additionally, we assessed the presence of horizontal pleiotropy by examining the Egger intercept (17). A *p*-value greater than 0.05 in this context was indicative of an absence of horizontal pleiotropy (23), suggesting that the instrumental variables used did not have pleiotropic effects that could bias the results. To investigate this phenomenon further, we utilized the MR-PRESSO global test to detect horizontal pleiotropy, where a genetic variant may influence multiple traits and complicate causal assessments. We also conducted heterogeneity tests on the instrumental variables we used. If these tests showed a *p*-value greater than 0.05, it meant that the heterogeneity wasn't significant and could be overlooked. All the Mendelian randomization (MR) analyses were carried out with the TwoSampleMR package in R (version 4.3.0).

Consequently, potential candidate metabolites implicated in the risk of PAD were selected based on the following criteria: (1) consistent associations across the five MR methods; (2) absence of detected pleiotropy; and (3) absence of high influence points identified by Leave-one-out analysis.

### 2.5 Reverse MR analyses

For our study, we extracted SNPs associated with PAD from GWAS summary statistics using a stringent significance threshold (*p*-value < 5 × 10^−8^). We then chose SNPs that were independent (with an r-squared value less than 0.001) within the European panel. These independent SNPs were utilized as instrumental variables (IVs) in Mendelian randomization (MR) analysis to investigate potential candidate metabolites.

## 3 Results

**Figure 2:**
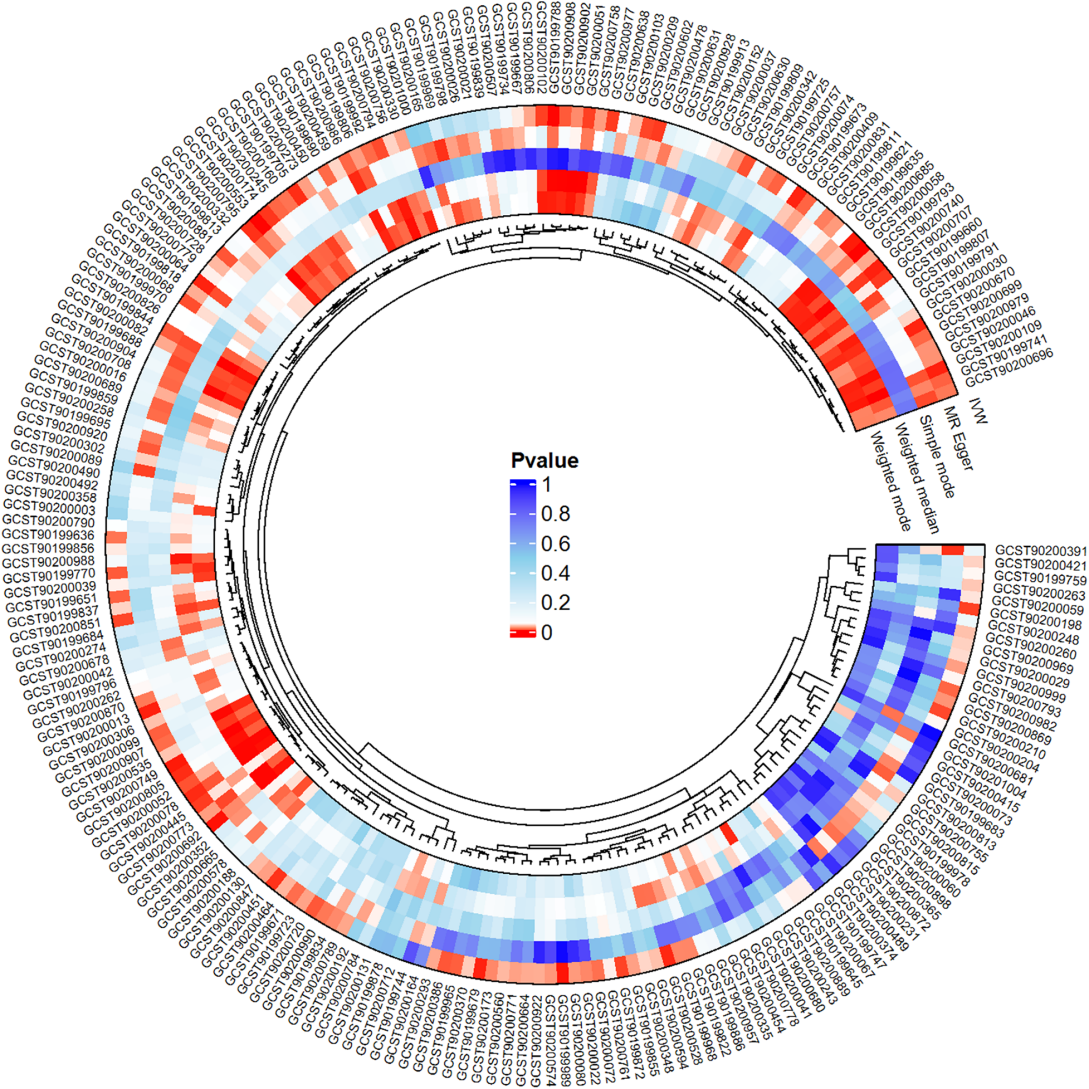
Circle map of the MR analysis results for metabolites and peripheral artery disease.

**Figure 3:**
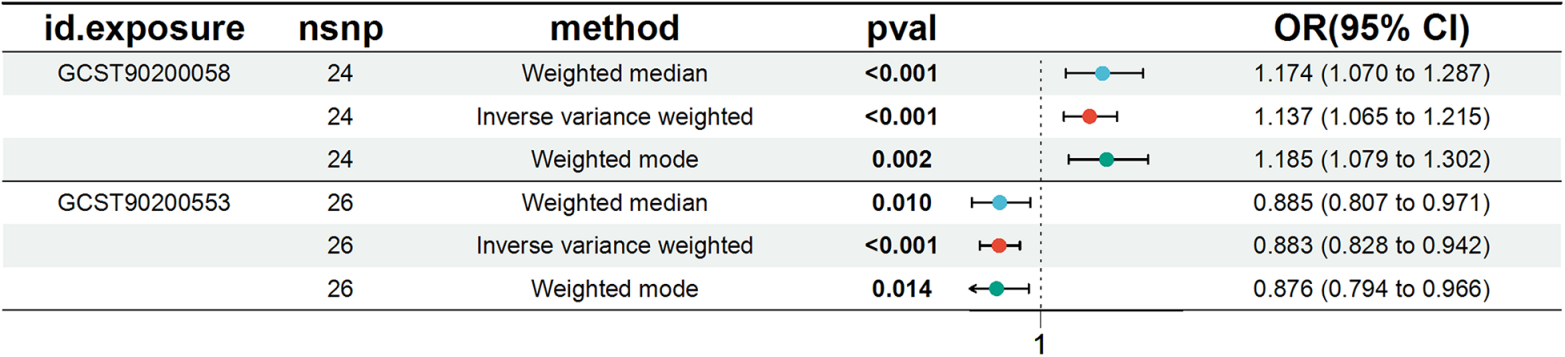
Forest plot for the causal effect of metabolites on the risk of peripheral artery disease derived from three methods (inverse variance weighted, weighted median, and weighted mode). OR, odds ratio; CI, confidence interval.

**Figure 4:**
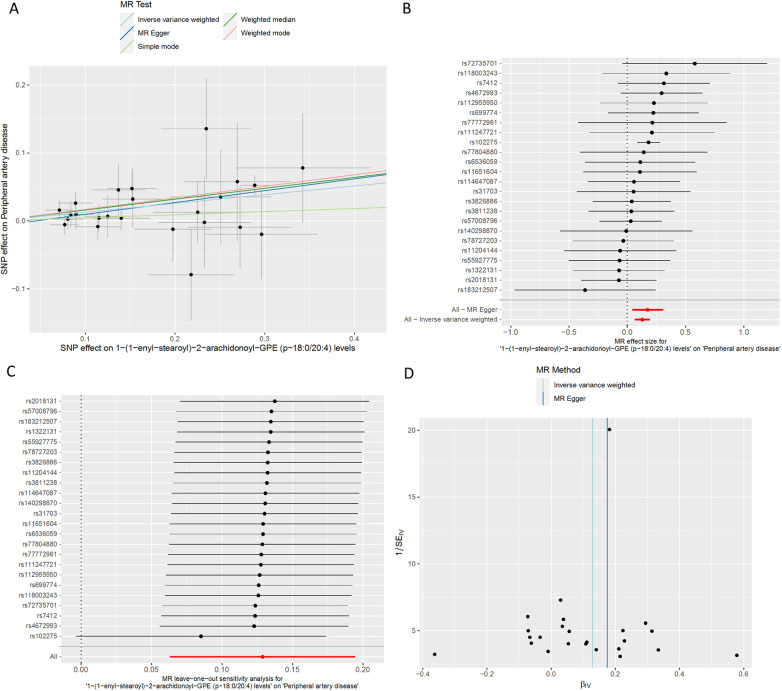
MR plots for the relationship of 1-(1-enyl-stearoyl)-2-arachidonoyl-GPE (p-18:0/20:4) with peripheral artery disease. **(A)** Scatter plot of SNP effects. **(B)** Forest plot presenting effect sizes estimated by individual and combined SNP MR analyses. **(C)** Leave-one-out plot. **(D)** Funnel plot.

**Figure 5:**
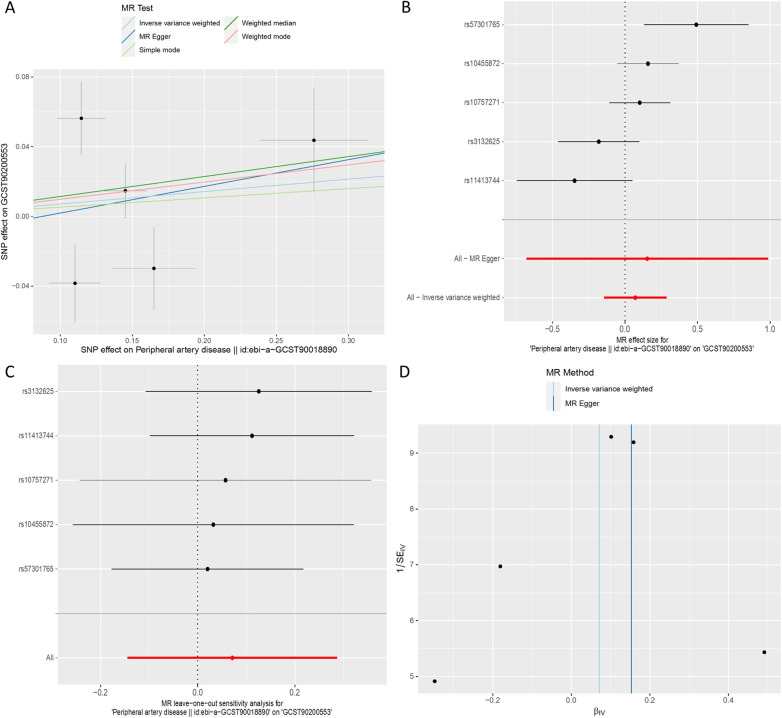
MR plots for the relationship of X-17653 with peripheral artery disease. **(A)** Scatter plot of SNP effects. **(B)** Forest plot presenting effect sizes estimated by individual and combined SNP MR analyses. **(C)** Leave-one-out plot. **(D)** Funnel plot.

We conducted a comprehensive Mendelian randomization (MR) analysis for each of the 1,400 metabolites under investigation. Subsequently, adhering to stringent IV selection criteria, we incorporated an extensive set of 34,127 Single Nucleotide Polymorphisms (SNPs) associated with Peripheral Artery Disease (PAD) into our analysis. From the initial pool, 215 blood metabolites were singled out based on a significance threshold of *p* < 0.05 in at least five MR analyses. A circular map visually represented these metabolites (Figure 2). Our selection process focused on identifying metabolites that consistently exhibited significant associations across all five methods, with a significance threshold of *p* < 0.05 in IVW analysis and a significance threshold of pFDR < 0.2. Moreover, if the heterogeneity test result yielded a *p*-value greater than 0.05, the influence of heterogeneity was deemed negligible. Ultimately, we finally detected two metabolites associated with peripheral artery disease (PAD), one known (1-(1-enyl-stearoyl)-2-arachidonoyl-GPE (p-18:0/20:4)) and one unknown (X-17653) (Figure 3). Levels of 1-(1-enyl-stearoyl)-2-arachidonoyl-GPE (p-18:0/20:4) in blood showed a significant association with PAD. Specifically, we observed a marked elevation in 1-(1-enyl-stearoyl)-2-arachidonoyl-GPE (p-18:0/20:4) levels among PAD patients compared to non-PAD patients, indicating a significant difference (IVW OR = 1.13, 95% CI, 1.06–1.21; *P* < 0.001) (Figure 4). This suggests a potential correlation between this metabolite's levels and PAD's occurrence and progression. Similarly, X-17653 levels significantly decreased in PAD patients (IVW OR = 0.88, 95% CI, 0.83–0.94; *P* < 0.001). The correlation between elevated X-17653 levels and reduced risk of PAD further supports the potential role of blood metabolites in the pathogenesis of PAD (Figure 5).

To enhance the robustness of our findings and account for potential confounding factors, we employed a comprehensive set of statistical methods in our analysis. These methods included MR-Egger regression, Weighted median, Simple mode, and Weighted mode, in addition to the standard IVW analysis. We also conducted multiple-effect and heterogeneity tests to validate our results further (Tables 1, 2). By utilizing these rigorous approaches, we aimed to minimize potential biases and strengthen the reliability of our research outcomes. The F statistics of the genetic instruments indicated the absence of weak instrument bias. MR-PRESSO (24) did not identify any potential outliers.

**Table 1:** Heterogeneity results from the Cochran's *Q* test of significant causal links between blood metabolites and peripheral artery disease.

Id. exposure	Outcome	Method	Q	Q_df	Q_pval
GCST90200058	Peripheral artery disease	MR egger	13.81	22	0.91
GCST90200058		Inverse variance weighted	14.39	23	0.92
GCST90200553		MR egger	17.46	24	0.83
GCST90200553		Inverse variance weighted	17.76	25	0.85

**Table 2:** Pleiotropy results from egger intercept analysis and MR presso.

Id. exposure	outcome	MR egger_intercept	pval	MR-presso global
GCST90200058	Peripheral artery disease	−0.007	0.452	0.871
GCST90200553		0.005	0.590	0.865

In the context of reverse causation, where genetic predisposition to PAD is considered as an exposure, we conducted MR analyses to investigate the causal impact of PAD on 1-(1-enyl-stearoyl)-2-arachidonoyl-GPE (p-18:0/20:4) (GCST90200058) and X-17653 (GCST90200553).

**Figure 6:**
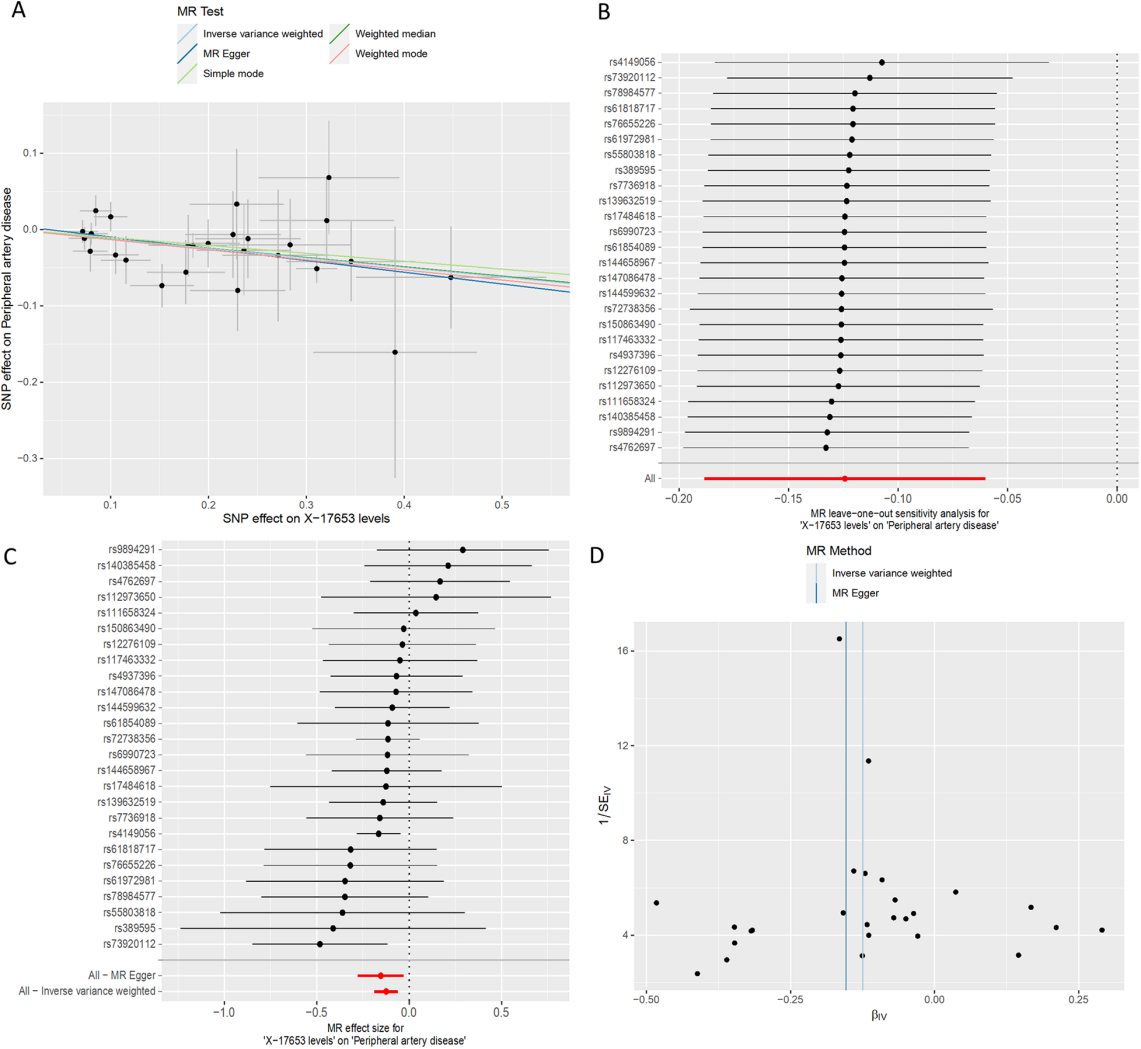
MR plots for the relationship of peripheral artery disease with X-17653. **(A)** Scatter plot of SNP effects. **(B)** Forest plot presenting effect sizes estimated by individual and combined SNP MR analyses. **(C)** Leave-one-out plot. **(D)** Funnel plot.

Across all MR methodologies employed (Table 3), no indication of a causal association between PAD and X-17653 (GCST90200553) was observed (IVW OR = 1.07, 95% CI, 0.87–1.33; *P* = 0.52) (Figure 6). The F statistics of the genetic instruments suggested no presence of weak instrument bias. MR-PRESSO analysis did not uncover any potential outliers. Consistent effect patterns were observed with the weighted median, weighted mode, simple mode, and MR-Egger methods. Leave-one-SNP-out analysis did not show any high leverage points exerting substantial influence.

**Table 3:** Reverse MR analysis results for 2 specific blood metabolites.

Id. exposure	OR (95%CI)	Pval	Q_pval_ IVW	Q_pval_MR. egger	Egger_intercept (pval)	MR-presso global
GCST90200058	1.16 (1.01–1.34)	0.036	0.203	0.123	0.788	0.251
GCST90200553	1.07 (0.87–1.33)	0.52	0.010	0.004	0.853	0.036

**Figure 7:**
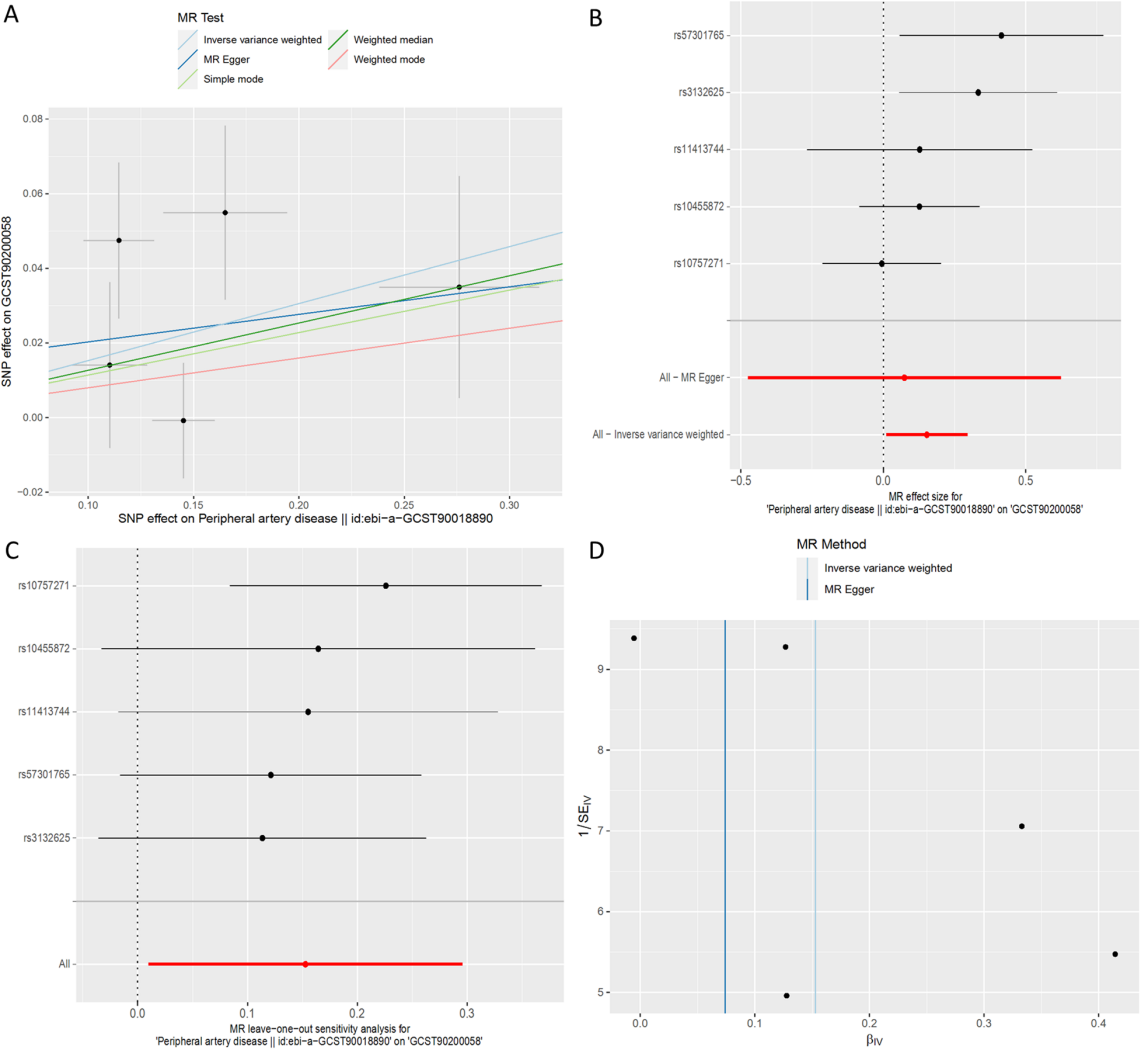
MR plots for the relationship of peripheral artery disease with 1-(1-enyl-stearoyl)-2-arachidonoyl-GPE (p-18:0/20:4). **(A)** Scatter plot of SNP effects. **(B)** Forest plot presenting effect sizes estimated by individual and combined SNP MR analyses. **(C)** Leave-one-out plot. **(D)** Funnel plot.

Utilizing inverse-variance weighted (IVW) methods, we observed a notable increase in the likelihood of PAD and 1-(1-enyl-stearoyl)-2-arachidonoyl-GPE (p-18:0/20:4) (GCST90200058) (IVW OR = 1.16, 95% CI, 1.01 to 1.34; *P* = 0.036) (Figure 7). However, other MR methodologies did not provide evidence of causal relationships (*P* > 0.05). The F statistics for the genetic instruments indicated the absence of weak instrument bias. MR-PRESSO analysis did not identify any potential outliers. Leave-one-SNP-out analysis did not reveal any high leverage points exerting significant influence.

## 4 Discussion

Our research findings confirm a causal relationship between two metabolites and PAD: 1-(1-enyl-stearoyl)-2-arachidonoyl-GPE (p-18:0/20:4) and X-17653. This research utilized available GWAS data and two-sample Mendelian randomization (MR) techniques to examine the causal link between PAD and blood metabolites. Rigorous sensitivity analyses were conducted to address confounding factors and enhance the robustness of our findings. Notably, this study represents an early endeavor to integrate metabolomic and genomic data, shedding light on the causal connections between serum metabolites and PAD.

Bidirectional Mendelian randomization analysis provides evidence supporting causal relationships between 1-(1-enyl-stearoyl)-2-arachidonoyl-GPE (p-18:0/20:4) and peripheral artery disease (PAD). Also known as PC[P-18:0/20:4(5Z,8Z,11Z,14Z)], 1-(1-enyl-stearoyl)-2-arachidonoyl-GPE (p-18:0/20:4) is a phosphatidylcholine (PC or GPCho) consisting of two distinct fatty acid chains: one comprising an 18-carbon atom acyl chain devoid of double bonds, and the other composed of a 20-carbon atom arachidonic acid chain with four double bonds. Arachidonic acid (25, 26), a polyunsaturated fatty acid, undergoes conversion into various inflammatory mediators, including prostaglandins (27), leukotrienes (28), and platelet-activating factors, capable of modulating vascular contraction and relaxation (29), thereby influencing blood pressure (4, 30). A recent study (31) found that 1-methylnicotinamide1-(1-enyl-stearoyl)-2-arachidonoyl-GPE was independently associated with the incidence of cardiovascular disease in fully adjusted models over a median period of 12.1 years (OR, 0.76; 95% CI, 0.65–0.89)). This suggests a potential mechanism whereby 1-(1-enyl-stearoyl)-2-arachidonoyl-GPE (p-18:0/20:4) may impact blood vessel tone and blood pressure by influencing arachidonic acid metabolism. Arul et al. (32) identified several key metabolites associated with acute ischemic stroke thrombi, including D-glucose, diacylglycerol, phytosphingosine, galabiosylceramide, glucosylceramide, and 4-hydroxynonenal. 1-(1-enyl-stearoyl)-2-arachidonoyl-GPE (p-18:0/20:4) is a plasmalogen involved in membrane composition and cell signaling. Compared to these metabolites, it may have a closer relationship with diacylglycerol and phytosphingosine because they all play important roles in lipid metabolism and cell membrane dynamics. Furthermore, the relationship between 1-(1-enyl-stearoyl)-2-arachidonoyl-GPE (p-18:0/20:4) and the glycolytic phenotype warrants further exploration. The glycolytic phenotype, characterized by increased glycolysis and reduced oxidative phosphorylation, is often associated with various pathological conditions, including cardiovascular diseases. Changes in plasmalogen levels may influence glycolytic activity, thereby contributing to the development of PAD. Further research is warranted to elucidate the precise molecular pathways linking 1-(1-enyl-stearoyl)-2-arachidonoyl-GPE (p-18:0/20:4) to PAD and to explore its therapeutic implications in the management of this condition.

X-17653 stands out among the metabolites associated with mitigated PAD risk, albeit its nature remains enigmatic, warranting further inquiry. Both 1-(1-enyl-stearoyl)-2-arachidonoyl-GPE (p-18:0/20:4) and X-17653 hold promise as prospective PAD biomarkers, potentially enhancing early diagnostic precision and offering innovative avenues for personalized therapeutic interventions and management strategies.

One of the principal strengths of our study lies in its comprehensive inclusion of a wide array of blood metabolites, totaling 1,400 in sum, for MR analysis. This renders our investigation the most extensive in probing the relationship between blood metabolites and PAD. Moreover, utilizing the MR design helps reduce the impact of confounding variables and reverse causation. Nevertheless, our study is not without its limitations. Firstly, the GWAS datasets utilized in our analysis were sourced from European populations, prompting inquiry into the generalizability of our findings to other ethnic groups, which warrants further exploration in future studies. Additionally, delving into the specific metabolic pathways associated with the identified metabolites exhibiting causal relationships constitutes an essential avenue for future research. Secondly, while MR methods facilitate the exploration of causal relationships, it is imperative to acknowledge the intricate interplay between genetic and environmental factors when interpreting these results. Genetic predispositions and environmental factors influence the regulation of blood metabolite levels, potentially impacting our understanding of their causal association with PAD. Lastly, while our MR analysis offers valuable insights into metabolites linked to PAD, it is crucial to emphasize that validating our study results requires rigorous randomized controlled trials, fundamental research endeavors, and future replication studies employing larger GWAS datasets focusing on PAD and metabolites.

## 5 Conclusion

In summary, this MR study identifies that 1-(1-enyl-stearoyl)-2-arachidonoyl-GPE (p-18:0/20:4) and X-17653 are associated with the risk of PAD. PAD is associated with increased 1-(1-enyl-stearoyl)-2-arachidonoyl-GPE (p-18:0/20:4) levels. This offers initial evidence regarding the influence of dysregulated blood metabolites on the risk of PAD.

## Data Availability

The original contributions presented in the study are included in the article/Supplementary Material, further inquiries can be directed to the corresponding author.
